# The Role of Food Peptides in Lipid Metabolism during Dyslipidemia and Associated Health Conditions

**DOI:** 10.3390/ijms16059303

**Published:** 2015-04-24

**Authors:** Chibuike C. Udenigwe, Kirsti Rouvinen-Watt

**Affiliations:** 1 Department of Environmental Sciences, Faculty of Agriculture, Dalhousie University, Truro, NS B2N 5E3, Canada; 2 Department of Plant and Animal Sciences, Faculty of Agriculture, Dalhousie University, Truro, NS B2N 5E3, Canada; E-Mail: Kirsti.Rouvinen-Watt@dal.ca

**Keywords:** bioactive peptides, hyperlipidemia, metabolic syndrome, cardiovascular disease, lipogenesis, hepatocytes, adipose tissue, hepatic steatosis

## Abstract

Animal and human clinical studies have demonstrated the ability of dietary food proteins to modulate endogenous lipid levels during abnormal lipid metabolism (dyslipidemia). Considering the susceptibility of proteins to gastric proteolytic activities, the hypolipidemic functions of proteins are possibly due, in part, to their peptide fragments. Food-derived peptides may directly modulate abnormal lipid metabolism in cell cultures and animal models of dyslipidemia. The peptides are thought to act by perturbing intestinal absorption of dietary cholesterol and enterohepatic bile acid circulation, and by inhibiting lipogenic enzymatic activities and gene expression in hepatocytes and adipocytes. Recent evidence indicates that the hypolipidemic activities of some peptides are due to activation of hepatic lipogenic transcription factors. However, detailed molecular mechanisms and structural requirements of peptides for these activities are yet to be elucidated. As hypolipidemic peptides can be released during enzymatic food processing, future studies can explore the prospects of combating metabolic syndrome and associated complications using peptide-rich functional food and nutraceutical products.

## 1. Dyslipidemia and Metabolic Syndrome

**Figure 1:**
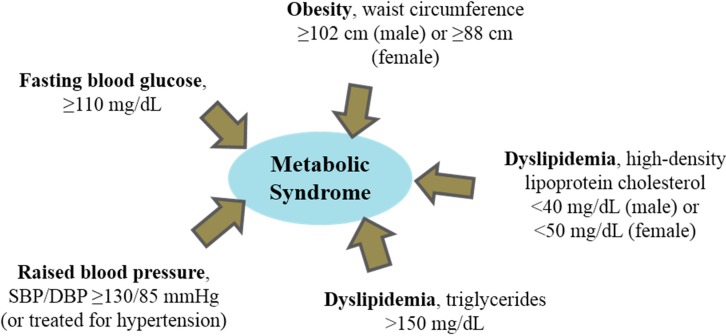
Dyslipidemia, abnormal endogenous lipid metabolism (including hyperlipidemia), is associated with metabolic syndrome. National Cholesterol Education Program Adult Treatment Panel III recommended that metabolic syndrome is diagnosed when an individual manifests three or more of the risk determinants [7].

Human lifestyle is gradually shifting towards an increase in the consumption of high energy diet (over-nutrition) and decrease in physical activity (sedentary lifestyle) [1]. This shift can lead to metabolic disorders and associated abnormalities often characterized by central obesity, hypertension, dyslipidemia and hyperglycemia, and other conditions such as non-alcoholic fatty liver disease [2,3]. These conditions are collectively termed metabolic syndrome (Figure 1) and, if uncontrolled, can lead to detrimental global public health outcomes. Metabolic syndrome indicators are also the known major risk factors of cardiovascular disease, a leading cause of death with estimated contribution to almost half of global mortality due to non-communicable diseases [4]. The molecular processes that occur in metabolic syndrome can lead to cardiovascular disease pathogenesis and involve risk factors such as oxidative stress and inflammation [2,5]. To date, changes in lifestyle (e.g., weight loss, physical activity and consumption of energy-restricted diet) have been recommended as primary strategies for controlling metabolic syndrome and related health problems. Moreover, therapeutic agents that target metabolic processes associated with the controllable risk factors have also been explored for the management of these health conditions [2]. Accordingly, nutritional and secondary metabolites in food (e.g., dietary fibers, polyunsaturated fatty acids, phytosterols, polyphenols, carotenoids and proteins) have demonstrated biological activities that can be applied in ameliorating abnormal metabolic processes related to metabolic syndrome [6]. These properties provide the opportunity for utilizing food for non-nutritional purposes in the promotion of human health and wellness.

## 2. Dietary Peptides and Hyperlipidemia

The consumption of food proteins has been found to modulate endogenous lipid profiles during dyslipidemia [8]. Soy β-conglycinin was recently reported to modulate *de novo* lipogenesis by inhibiting hepatic fatty acid synthase mRNA expression and enzymatic activity, with concomitant decrease observed in the levels of hepatic lipids (triglycerides, TG; total cholesterol, TC; phospholipids, PL) and liver weight in OLEFT rats with non-alcoholic fatty liver disease [9]. Hypolipidemic activities have also been observed for squid homogenate [10], chicken protein extract [11], soy proteins [12] and fermented milk [13] in different dyslipidemic rat models. Moreover, a randomized controlled double-blind crossover trial with 68 human subjects demonstrated that feeding 25 g lupin protein (incorporated into rolls, bread, spread, sausage) per day for 28 days lowered plasma lipids (TG, TC, low-density lipoprotein [LDL] cholesterol); the effects were found to be more pronounced in subjects with severe hypercholesterolemia [14]. Although phytochemicals present in protein extracts can contribute to bioactivity, hypolipidemic effects (decrease in abdominal fat, liver weight and free fatty acids, FFA) have also been attributed to protein fractions [12]. There is no evidence of a direct interaction of native dietary proteins with physiological lipidome during dyslipidemia. On one hand, arginine was suggested to be the contributor of the hypolipidemic function of the proteins due to its role as endogenous precursor of nitric oxide, which can interact with lipoprotein metabolism during dyslipidemia [14]. Although this mechanism is plausible, arginine residues of dietary proteins do not have similar bioavailability as the free amino acid form typically used in control diets. Furthermore, the peptide bonds of dietary proteins are susceptible to extensive hydrolysis by gastric and brush border protease and peptidase activities. Consequently, the hypolipidemic effects of dietary proteins are likely due to their peptide fragments, in addition to the possible contribution of their arginine residues. In fact, an arginine-containing oligopeptide (KRES) was found to lose its hypolipidemic activity when the order of amino acids was changed to KERS [15], indicating that the intact peptide structure (not the amino acid/arginine residues) was responsible for its activity in ApoE-null mice with dyslipidemia. Moreover, hydrolyzed casein (and not the intact protein) was found to possess hypolipidemic activities in obese-prone male C57BL/6J mice [16]. Furthermore, soy peptides decreased TG accumulation in yeast through the downregulation of lipogenic *DGA1* mRNA compared to treatments with proteins or amino acid mixture of similar amino acid composition [17]. These findings support the direct hypolipidemic effects of food protein-derived peptides.

The physiological processes that constitute metabolic dyslipidemia (e.g., elevated endogenous TG, TC, LDL cholesterol, and decreased HDL cholesterol) have been modulated by dietary food protein hydrolysates and peptides in cellular systems and animal models. The mechanisms of hypolipidemic activity of food protein hydrolysates and peptides have been discussed [18]. The peptides are thought to act by disrupting the micellar solubility and absorption of dietary cholesterol, altering enterohepatic bile acid circulation and elevating cholesterol catabolism, and regulating lipogenic proteins and genes. Moreover, high yields of food-derived hypolipidemic peptides (e.g., IIAEK, VPDPR, DPR) have been successfully expressed in *Escherichia coli*, and the resulting peptides demonstrated lipid-lowering activities when fed to rats [19]. This would encourage industry scale production of the peptides for future commercial applications. The structure of peptides may determine their bioavailability and tissue distribution (e.g., hydrophobic peptide uptake in adipocytes), but there is a dearth of clearly-defined understanding of structure-function relationships for hypolipidemic peptides. Recent studies have corroborated earlier findings on the modulation of lipid profiles with dietary food protein hydrolysates and peptides in animal models of dyslipidemia [20,21,22,23]. The present paper provides an update on the molecular mechanism of peptide-induced hypolipidemia, and use of the peptides in controlling metabolic disorders and related health conditions. Table 1 shows some hypolipidemic peptides of known structures and their proposed mechanisms of action.

**Table 1:** Peptide sequences with hypolipidemic activities **.

Peptide	Source	Activity/Mechanism	Reference
KNPQLR	Soybean β-conglycinin	Binding the active site and inhibition of FAS activity *in vitro* by interacting with FAS thioesterase domain/activity	[24]
EITPEKNPQLR			
RKQEEDEDEEQQRE			
LPYPR	Soybean proteins (glycinin)	HMGCoAR inhibition *in vitro* (LPYPR); disruption of cholesterol micellar solubility *in vitro*; upregulated lipogenic genes *CYP51*, *LDLR*, *LPL* and *CYP7A1* resulting in reduction in plasma VLDL-C, TG, but increased plasma TC with low fecal sterol excretion in diet-induced hyperlipidemic mice	[23]
WGAPSL			
WE	Synthetic	Direct binding and transactivation of PPARα; increased expression of PPARα-responsive genes of fatty acid metabolism, *FATP4*, *ACS*, *CPT1* and *ACOX*, and reduced intracellular cholesterol and TG levels in hepatic cell culture	[25]
KRES	Synthetic	Increased plasma HDL-C and reduced atherosclerosis (in addition to its antioxidative activities) in apoE null mice; no known mechanism	[15]
KDW	Synthetic	Increased plasma HDL-C and decreased plasma LDL-C, TC, TG and atherogenic index in diet-induced hyperlipidemic rats; no known mechanism	[22]
YPFVV (soymorphin-5)	Soybean protein (β-conglycinin)	Decreased plasma and liver TG, and liver weight; increased plasma adiponectin, hepatic adiponectin receptor and PPARα expression leading to upregulation of genes involved in fatty acid β-oxidation in diabetic KKAy mice	[26]
HIRL (β-lactotensin)	Milk protein (β-lactoglobulin)	Decreased serum LDL-C and TC in diet-induced hyperlipidemic mice mediated by neurotensin (NT2) and dopamine (D2) receptors, and stimulated bile acid secretion	[27]

** A comprehensive list of other food-derived hypolipidemic peptides and their mechanisms of action have been recently reviewed by Howard & Udenigwe [18]; HMGCoAR, 3-hydroxy-3-methylglutaryl coenzyme A reductase; VLDL-C, very low-density lipoprotein cholesterol; HDL-C, high-density lipoprotein cholesterol; LDL-C, low-density lipoprotein cholesterol; TG, triglycerides; TC, total cholesterol; PPAR, peroxisome proliferator-activated receptor; *FATP4*, fatty acid transport protein 4 gene; *ACS*, acyl-CoA synthetase gene; *CPT1*, carnitine palmitoyltransferase 1 gene; *ACOX*, acyl-CoA oxidase gene.

## 3. Intestinal Functions of Hypolipidemic Peptides

**Figure 2:**
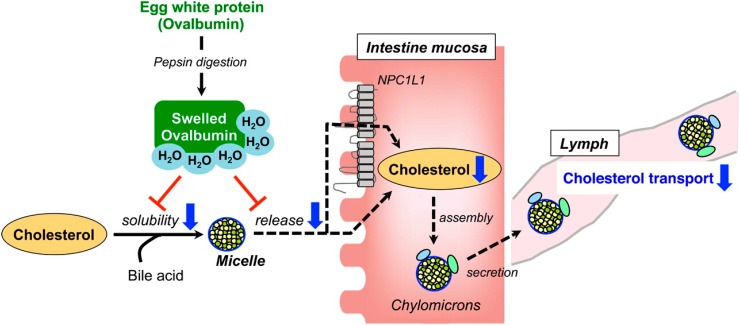
The role of egg white protein digested with pepsin on intestinal and lymphatic uptake of dietary cholesterol in rats. Reprinted from [30] with permission form American Chemical Society, copyright 2014.

The intestine is thought to be a major site of action of hypolipidemic peptides. Dietary cholesterol is incorporated into micelles with PL and bile salts prior to intestinal uptake [28]. Food protein hydrolysates and peptides are known to disrupt the incorporation of cholesterol into simulated micelles; see review [18]. This activity was recently reported for egg white and cowpea protein hydrolysates and thought to be a plausible mechanism of the hypolipidemic activity of the peptide products [20,29]. As shown in Figure 2, alteration of intestinal cholesterol uptake by the pepsin-digested egg white proteins resulted in increased fecal excretion of neutral steroids and decreased lymphatic cholesterol transport in rats possibly due to inhibition of chylomicron assembly [30]. Although this effect is based on physical disruption of intestinal assembly and release of dietary cholesterol, there is a possible involvement of dietary lipid transport mediators, e.g., intestinal Niemann-Pick C1-like 1 (NPC1L1) protein. A recent study reported that mRNA expression of NPC1L1 and fatty acid translocase (CD36/FAT) are decreased in the jejunum of rats that received peptide-containing squid muscle protein homogenate diet resulting in a healthy plasma lipid profile [10]. It is not apparent if the effect on the intestinal lipid transport gene expression is mediated by direct interaction with the peptides, or due to reduced need and induction of the transport proteins with the lower amounts of intestinal cholesterol (or fatty acids).

Endogenous cholesterol is metabolized in hepatocytes to fat-soluble bile acids, which are secreted in the duodenum as water-soluble bile salts, and later reabsorbed through enterohepatic circulation [28]. This pathway has presented an opportunity for the use of dietary agents, including protein hydrolysates and peptides, in elevating endogenous cholesterol metabolism through the removal of intestinal bile acids. Hydrophobic bioactive peptides are known to bind bile acids possibly due to hydrophobic interaction with the tetracyclic ring structures [18], contributing to increased fecal excretion of the sterols. However, low fecal sterol excretion and increased plasma cholesterol levels have been observed for hydrophobic soy protein-derived pentapeptides, LPYPR and WGAPL, although the peptides were found to decrease plasma VLDL cholesterol and TG levels; the beneficial effects of the peptides were suggested to be mediated through other processes [23]. Apart from hydrophobicity, there is a dearth of information on structure-function relationships for intestinally-active hypolipidemic peptides, but amino acid residues with strong cationic properties at physiological pH (e.g., Lys and Arg) can also be explored for the ability to interact with the carboxylic group of bile acids and in enhancing sequestration and fecal excretion. Moreover, low digestibility of proteins and peptides (*i.e.*, resistant to gastric proteolysis) was found to be important for gastric hypolipidemic activity [30]. Bulky amphipathic peptides (e.g., soy protein-derived peptide LPYPR) can also be explored for the ability to penetrate and disrupt the micellar structure and possibly reduce physiological absorption of dietary cholesterol. This approach to modulating dyslipidemia is promising since the peptides do not need to be absorbed into circulation, a factor that currently impedes the application of dietary bioactive peptides as functional ingredients for human health promotion [31].

## 4. Adipocytic Functions of Hypolipidemic Peptides

Peptides are known to exert hypolipidemic effects in adipose tissues by inhibiting fatty acid synthase (FAS), pre-adipocyte differentiation and elevating expression of PPARγ co-activator-1α, uncoupling protein-1, carnitine palmitoyltransferase-1/2, and medium chain and long chain acyl-CoA dehydrogenases, which promote fatty acid oxidation; see review [18]. The α and α’ subunits of soybean β-conglycinin digested with Alcalase were found to exhibit hypolipidemic effects by FAS activity inhibition *in vitro* and by decreasing lipid accumulation (pre-adipocyte differentiation) in human subcutaneous adipose tissue culture [32]. Peptides identified in the FAS-inhibiting soybean β-conglycinin hydrolysate (KNPQLR, EITPEKNPQLR and RKQEEDEDEEQQRE) were synthesized and found to act by directly binding and inhibiting the thioesterase domain of FAS, resulting in dose-dependent inhibition of lipid droplet accumulation in cultured 3T3-L1 adipocytes [24]. Similarly, soybean protein hydrolysate produced with pepsin was found to dose-dependently increase the expression of fatty acid-binding protein gene (*aP2*), an adipogenic marker gene, and decrease the expression of preadipocyte factor-1 mRNA (*Pref-1*) with concomitant increase in adiponectin secretion in cultured adipocytes [33]. The effect observed in the study was thought to be mediated by PPARγ upregulation as the hypolipidemic activity was lost in the presence of a PPARγ antagonist. In order to directly interact with adipocytes and exert these activities, dietary peptides must be absorbed and transported to the adipose tissue. Information on the physiological distribution of dietary peptides especially in adipocytes is lacking. Future studies should focus of tracking the hypolipidemic soybean peptides *in vivo* to ascertain if they are responsible for the physiological effects of the dietary proteins. The direct interaction of peptides with adipocytes is plausible considering earlier reports of anti-obesity effects (decreased body/adipose tissue masses) and molecular events (gene regulation that can increase mitochondrial fatty acid oxidation and uncoupling of oxidative phosphorylation) triggered by dietary hydrolysates of salmon protein and casein (compared to intact casein) in white adipose tissue of Wistar Hannover GALAS rats and C57BL/6J mice, respectively [16,34].

## 5. Hepatic Functions of Hypolipidemic Peptides

Hepatocytes play a central role in lipid metabolism, and food protein hydrolysates and peptides have demonstrated several effects on hepatic lipogenic genes and proteins resulting in the modulation of endogenous lipid profiles. As recently reviewed [18], food peptides have modulated the activity or expression of lipogenic enzymes (FAS, malic enzyme, phosphatidate phosphohydrolase, glucose 6-phosphate dehydrogenase, acyl-CoA cholesterol acyl transferase [ACAT], stearoyl-CoA desaturase-1 [SCD1], Δ5/Δ6-desaturases), decreased LDL/VLDL synthesis (apoB100 inhibition), and elevated the expression of fatty acid oxidation proteins (PPARα, adiponectin receptor, carnitine palmitoyltransferase, acyl-coenzyme A oxidase, uncoupling proteins) and cholesterol degrading proteins (LDL receptor, 7α-hydroxylase) in cell culture and animal models of dyslipidemia. A possible mechanism for the effects on VLDL was recently proposed to be related to TG metabolism. Hepatic expression of microsomal TG transfer protein (MTP) mRNA was found to be decreased in rats treated with squid protein homogenate [10]. Hepatic MTP functions in VLDL assembly and secretion and its downregulation was thought to have contributed to the observed hypolipidemic events in the liver cells. Moreover, other peptides exhibited *in vitro* inhibition of the activity of 3-hydroxy-3-methylglutaryl coenzyme A reductase (HMGCoAR), which catalyzes the rate-limiting step of endogenous cholesterol biosynthesis [29,35]. This activity is expected to modulate dyslipidemia by lowering hepatic and plasma cholesterol levels. Moreover, the hypolipidemic activity of food proteins (or their peptide fragments) has been related to their effects on lipogenic transcription factors. For instance, dietary soybean β-conglycinin and chicken protein fraction were found to downregulate PPARγ2 protein and *Srebf1* gene, respectively, in diet-induced hyperlipidemic animals [11,36]. Moreover, detailed hepatic microarray analysis has shown a >2-fold change in the expression of PPAR signaling pathway genes in rats that consumed salmon protein hydrolysate [34]. Furthermore, dipeptide WE was also found to reduce lipid accumulation in hepatocytes through PPARα agonist activity and activation of PPARα-responsive genes including *FATP4*, *ACS*, *CPT1* and *ACOX* in hepatocytes [25]. The PPARα-mediated signaling events will particularly promote hepatic fatty acid metabolism and energy expenditure whereas upregulation of SREBP gene would enhance cholesterol metabolism. Similar PPARα-mediated modulation of lipogenic gene expression was reported in diabetic KKAy mice that received soymorphin-5 (YPFVV), a pentapeptide derived from the β-subunit of soybean β-conglycinin [26]. The lipogenic gene regulation induced by soymorphin-5 was associated with hypolipidemic effects including decrease in plasma and liver TG, decrease in liver weight and increase in plasma adiponectin in the diabetic mice [26]. Despite the prospects, there is still a knowledge gap on interaction of the food-derived peptides with upstream molecular events associated with the transcription factors. Recently, the SREBP2-mediated effect of lupin peptides was suggested to be due to the activation of phosphoinositide 3 kinase (PI3K)/protein kinase B (Akt) pathways, which led to increased expression of hepatic LDLR proteins and LDL uptake in the cultured cells [35]. As observed in the study, HMGCoAR protein (coded by a SREBP-responsive gene) was elevated in hepatocytes due to SREBP2 activation [35], but the cellular enzymatic activity was not evaluated to ascertain if the hepatic enzyme can be directly inhibited by the lupin peptides (as demonstrated *in vitro* in the study) towards inhibition of *de novo* lipogenesis.

It is important to note that the effects on transcription factors that control lipid metabolism were mostly observed with food peptide mixtures (protein hydrolysates or fractions), which makes it challenging to identify particular bioactive principles or decipher the structural basis of activity of the peptides. One possible mechanism involved in the modulatory effects could be due to the direct interaction of peptides with the transcription factors of lipid metabolism. Recently, binding modeling assay showed that dipeptide WE bound PPARα through H-bonding with its Glu286, Asn219 or Met220 residues leading to conformational changes that increased the proximity of a coactivator peptide [25]. In addition to PPARα transactivation, WE was reported to have antagonist activity against PPARγ, which is involved in lipogenesis [37]. Therefore, the dipeptide can simultaneously induce the inhibition of *de novo* lipogenesis and activation of lipid uptake and degradation in hepatocytes. Moreover, it is also possible that hypolipidemic peptides within the hydrolysates can interact with one another to induce pronounced hypolipidemic effects. Although hydrophobicity is a desirable peptide functionality for some hypolipidemic activities (e.g., bile acid binding), a hydrophilic peptide fraction (that yielded hypolipidemic dipeptide KA, VK, SY) was found to retain the activity of its parent soy protein hydrolysate by decreasing TG synthesis and apoB100 secretion in cultured hepatocytes [38]. Intestinal absorption and cellular uptake is required for the peptides to exert their activities in hepatocytes, and this is particularly plausible with the hypolipidemic dipeptides WE, KA, VK and SY. The small sizes of the dipeptides can make them resistant to endogenous proteolytic inactivation, and possibly promote their cellular uptake through proton-dependent peptide transporters (hPepT1, hPepT2).

## 6. Conclusions

Based on current literature, it is expected that the hypolipidemic effects of dietary food proteins could be due to the peptides released after enzymatic hydrolysis in the gastrointestinal tract. These effects could be centrally mediated by direct interaction of the peptides with transcription factors and enzymatic activity related to endogenous lipid metabolism especially in hepatocytes and adipocytes. Moreover, physical disruption of dietary cholesterol absorption and enterohepatic bile acid reabsorption by the peptides can play key roles in endogenous cholesterol homeostasis. The hypolipidemic peptides hold promise for use in suppression of hepatic lipid synthesis and accumulation especially in health conditions such as hepatic steatosis (non-alcoholic fatty liver disease). Most evidence presented to date is based on *in vitro*, cellular and animal studies. Therefore, human clinical studies are needed to validate bioactivity in order to make conclusive statements on the application of hypolipidemic food-derived peptides.
